# Unveiling the Intricacies: A Comprehensive Review of Magnetic Resonance Imaging (MRI) Assessment of T2-Weighted Hyperintensities in the Neuroimaging Landscape

**DOI:** 10.7759/cureus.54808

**Published:** 2024-02-24

**Authors:** Rishabh Dhabalia, Shivali V Kashikar, Pratap S Parihar, Gaurav V Mishra

**Affiliations:** 1 Radiodiagnosis, Jawaharlal Nehru Medical College, Datta Meghe Institute of Higher Education & Research, Wardha, IND

**Keywords:** neurological diseases, artificial intelligence, multidisciplinary approach, mri assessment, neuroimaging, t2-weighted hyperintensities

## Abstract

T2-weighted hyperintensities in neuroimaging represent areas of heightened signal intensity on magnetic resonance imaging (MRI) scans, holding crucial importance in neuroimaging. This comprehensive review explores the T2-weighted hyperintensities, providing insights into their definition, characteristics, clinical relevance, and underlying causes. It highlights the significance of these hyperintensities as sensitive markers for neurological disorders, including multiple sclerosis, vascular dementia, and brain tumors. The review also delves into advanced neuroimaging techniques, such as susceptibility-weighted and diffusion tensor imaging, and the application of artificial intelligence and machine learning in hyperintensities analysis. Furthermore, it outlines the challenges and pitfalls associated with their assessment and emphasizes the importance of standardized protocols and a multidisciplinary approach. The review discusses future directions for research and clinical practice, including the development of biomarkers, personalized medicine, and enhanced imaging techniques. Ultimately, the review underscores the profound impact of T2-weighted hyperintensities in shaping the landscape of neurological diagnosis, prognosis, and treatment, contributing to a deeper understanding of complex neurological conditions and guiding more informed and effective patient care.

## Introduction and background

T2-weighted hyperintensities have emerged as a crucial focal point within the field of neuroimaging. These enigmatic radiological findings, as revealed through magnetic resonance imaging (MRI), have captured the attention of researchers, clinicians, and medical practitioners alike. Their importance lies in their potential to serve as sensitive markers for various neurological disorders and to shed light on the underlying pathophysiological processes. Understanding T2-weighted hyperintensities is essential for accurate diagnosis, prognosis, and the development of effective treatment strategies in neurology [1].

The intricacies of T2-weighted hyperintensities, which manifest as areas of increased signal intensity on T2-weighted MRI images, have far-reaching implications for diverse neurological conditions, such as Alzheimer’s disease, multiple sclerosis (MS), stroke, and vascular dementia. Their presence can indicate areas of abnormal tissue, inflammation, demyelination, or ischemia, making them invaluable for disease detection and monitoring [2].

The purpose of this comprehensive review article is to delve deep into the world of T2-weighted hyperintensities in neuroimaging. We aim to provide a thorough exploration of these hyperintensities, from their definitions and underlying causes to the advanced neuroimaging techniques used for their assessment. By offering a comprehensive understanding, we aim to elucidate the clinical significance of T2-weighted hyperintensities and their role as diagnostic and prognostic markers. Through a meticulous literature analysis, we will categorize and discuss different types of T2-weighted hyperintensities, distinguishing between white and gray matter hyperintensities and their unique features. Furthermore, we will highlight the challenges and pitfalls associated with their assessment, including issues related to image artifacts and inter-rater variability.

## Review

Understanding T2-weighted hyperintensities

Definition and Characteristics

T2-weighted hyperintensities represent a distinctive feature in MRI that appears as areas of heightened signal intensity on T2-weighted images. These areas differ from the surrounding tissues regarding signal intensity, and their characteristics are vital for their identification and differentiation from typical brain structures [3].

Increased signal intensity: T2-weighted hyperintensities are characterized by their elevated signal intensity when visualized on T2-weighted MRI images. This heightened signal intensity causes them to appear brighter compared to the surrounding brain tissue, resulting in a stark visual contrast. This characteristic is fundamental for detecting and differentiating from typical brain structures, providing a clear and easily identifiable marker within the imaging data [4].

Location variability: T2-weighted hyperintensities can manifest in diverse brain regions, and their location is not restricted to specific anatomical areas. Their variability in location adds complexity to their interpretation, as they can be found in various brain regions. Moreover, these hyperintensities may exhibit distinct morphological patterns, such as focal lesions, diffuse areas, or linear streaks, depending on the underlying pathological processes. The diverse presentation of hyperintensities underscores their multifaceted nature in neuroimaging [5].

Hyperintensity grading: T2-weighted hyperintensities exhibit a spectrum of signal intensity, and their degree of hyperintensity can vary significantly. Radiologists and clinicians often use grading systems to categorize hyperintensities based on their intensity, ranging from mild to moderate to severe. This grading system reflects the differences in the underlying pathological changes that give rise to these hyperintensities. The extent of hyperintensity can offer insights into the severity and stage of the associated neurological condition [6].

Contrast with normal brain tissue: One of the defining characteristics of T2-weighted hyperintensities is their stark contrast with adjacent or normal brain tissue. This inherent contrast facilitates their identification and evaluation during the neuroimaging process. Differentiating these hyperintensities from the surrounding brain structures is essential for accurate diagnosis and plays a pivotal role in highlighting regions of abnormality within the brain. This contrast is a cornerstone of the clinical utility of T2-weighted hyperintensities, aiding in their recognition and interpretation by healthcare professionals [7].

Clinical Relevance and Significance

Diagnostic utility: T2-weighted hyperintensities are indispensable diagnostic markers for evaluating neurological disorders. Their presence and characteristics are pivotal in identifying conditions such as MS, vascular dementia, and stroke. For instance, detecting hyperintensities in MS is a critical diagnostic criterion, enabling clinicians to confirm the presence of demyelination and inflammation within the central nervous system. By highlighting regions of abnormality within the brain, T2-weighted hyperintensities play a central role in accurately diagnosing these conditions [8].

Prognostic value: Beyond their diagnostic significance, T2-weighted hyperintensities hold substantial prognostic value. Their presence, extent, and evolution over time provide clinicians with critical insights into disease progression, enabling them to predict future patient outcomes. In conditions like stroke and vascular disorders, the severity and distribution of hyperintensities can correlate with long-term functional outcomes and the likelihood of recurrent vascular events. This prognostic information helps healthcare providers tailor treatment strategies and interventions, ultimately improving patient care and optimizing therapeutic approaches [9].

Monitoring disease activity: T2-weighted hyperintensities are pivotal in monitoring disease activity and treatment response, particularly in conditions like MS. These hyperintensities can change in size and distribution over time, reflecting ongoing disease activity. By regularly assessing the evolution of these hyperintensities through follow-up MRI scans, clinicians can gauge the effectiveness of therapy and adjust treatment plans accordingly. This dynamic monitoring is crucial for optimizing patient care, as it allows for timely interventions and modifying treatment strategies to control disease activity and progression better [10].

Research insights: T2-weighted hyperintensities serve as valuable endpoints in clinical research studies, providing researchers with insights into disease mechanisms and treatment outcomes. These hyperintensities are used as objective measures to evaluate the efficacy of novel interventions, such as disease-modifying therapies, in MS. Researchers can quantify changes in hyperintensity burden and distribution to assess the impact of treatments on disease progression. Additionally, the presence and characteristics of T2-weighted hyperintensities in research cohorts contribute to a deeper understanding of the pathophysiology of neurological conditions and guide the development of targeted therapeutic approaches. This research-driven approach is instrumental in advancing the field of neurology and improving patient care [11].

Common Underlying Causes

Ischemia and infarction: T2-weighted hyperintensities can arise due to ischemia and infarction, where areas of the brain experience reduced blood flow or complete deprivation of oxygen and nutrients. This condition is notably observed in ischemic strokes, leading to hyperintense regions forming on T2-weighted MRI scans. Due to edema and cellular changes, the increased water content in these infarcted areas results in the bright appearance of hyperintensities. These hyperintensities serve as a crucial diagnostic marker in stroke assessment, aiding in the localization and evaluation of infarcted brain tissue [12].

Inflammation and demyelination: Inflammatory and demyelinating disorders, such as MS, can give rise to T2-weighted hyperintensities. In these conditions, the immune system’s inflammatory response targets the myelin sheath, resulting in demyelination and disruption of nerve conduction. These pathological changes lead to heightened water content and increased signal intensity, manifesting as hyperintensities on T2-weighted images. Identifying and characterizing these hyperintensities is pivotal for diagnosing and monitoring such disorders, allowing healthcare professionals to assess disease activity and treatment response [13].

Vascular abnormalities: Various abnormalities, including microangiopathies and venous infarctions, can produce T2-weighted hyperintensities in neuroimaging. Microangiopathies, often associated with small vessel disease, lead to changes in the blood-brain barrier and increased water content in the brain tissue. This can result in the presence of hyperintensities on MRI scans. Venous infarctions, which are caused by impaired venous outflow and subsequent tissue congestion, can also lead to the formation of hyperintensities. Recognizing these hyperintensities is essential for diagnosing vascular abnormalities and understanding their clinical implications [14].

Age-related changes: T2-weighted hyperintensities are observed in normal aging and are often referred to as age-related white matter changes. These changes are particularly prevalent in the elderly population and manifest as hyperintensities within the brain’s white matter. While these hyperintensities are considered a part of the aging process, their presence can still have clinical significance, as their extent and location may influence cognitive function and the risk of age-related neurological conditions. Accurate differentiation between age-related changes and pathological hyperintensities is critical for clinical assessment [15].

Tumor-related changes: Brain tumors, both primary and secondary, can lead to the formation of T2-weighted hyperintensities. These hyperintensities are often associated with peritumoral edema due to increased intracranial pressure and tumor growth. The accumulation of fluid and cellular changes in the vicinity of the tumor results in hyperintense regions on T2-weighted MRI scans. The recognition and characterization of these hyperintensities are instrumental in localizing and assessing the extent of tumor-related changes, guiding treatment decisions, and monitoring the response to therapies [16].

Infection and inflammatory conditions: Infectious diseases, such as encephalitis or other inflammatory processes within the brain, can give rise to T2-weighted hyperintensities. In the case of encephalitis, the immune response to viral or bacterial agents may lead to increased water content and inflammation in the affected brain regions. This, in turn, results in the appearance of hyperintensities in neuroimaging. Identifying these hyperintensities is crucial for diagnosing infectious and inflammatory conditions, enabling timely intervention and appropriate treatment strategies. The presence and characteristics of these hyperintensities can also aid in monitoring disease activity and response to therapy [17].

Types of T2-weighted hyperintensities

White Matter Hyperintensities (WMH) and Gray Matter Hyperintensities

WMH form a significant category of hyperintense regions in T2-weighted neuroimaging. These can be further categorized into two distinct groups [2]. Age-related changes encompass WMH frequently observed in the aging brain, with a higher prevalence among older individuals. These changes are often referred to as “age-related white matter changes” or “leukoaraiosis” and result from various factors such as reduced blood flow, microvascular alterations, and the accumulation of small vascular lesions. Without neurological symptoms, these age-related WMH are typically considered incidental and benign, although they may still contribute to cognitive decline in the elderly [18]. Pathological conditions involve the presence of WMH in specific medical contexts. In cases of vascular dementia, WMH is commonly found because of cerebrovascular disease and small vessel pathology. The extent and severity of these hyperintensities are closely linked to the cognitive impairment seen in patients with vascular dementia. Furthermore, WMH in MS indicate demyelination and inflammation, playing a pivotal role in diagnosing and monitoring disease progression [19]. Gray matter hyperintensities represent a distinct category of T2-weighted hyperintensities that occur in gray matter regions of the brain. Their presence may signal underlying pathological conditions, such as cerebral microbleeds, infections, or cortical laminar necrosis. While less common than WMH, they can have significant clinical implications [20].

Differential Diagnosis and Distinguishing Features

Location: Discriminating between white matter and gray matter hyperintensities is primarily based on their distinct anatomical locations within the brain. WMH are typically found in the deep white matter regions and periventricular areas, rich in myelinated nerve fibers. In contrast, gray matter hyperintensities are located within the gray matter structures of the brain, encompassing regions where neuronal cell bodies and synapses are concentrated. This difference in location is instrumental in their differentiation and can guide clinicians in understanding their potential clinical significance [21].

Morphology: The morphology or appearance of white and gray matter hyperintensities on MRI images can vary significantly. WMH frequently exhibit well-defined, patchy, or confluent areas of increased signal intensity. They may appear as discrete, round, or oval lesions or as more extensive brightness areas, often with a periventricular distribution. In contrast, gray matter hyperintensities tend to have a more irregular and less well-defined morphology. They may appear as small, irregularly shaped regions of hyperintensity within gray matter structures. Recognizing these distinct morphological patterns is valuable in distinguishing between the two types of hyperintensities [22].

Clinical correlation: The clinical context in which hyperintensities are observed is critical to their differentiation. For example, in older individuals, WMH are often associated with age-related changes in the brain, commonly referred to as leukoaraiosis. These age-related white matter changes may be benign and related to cognitive decline in the elderly. In such cases, the clinical correlation is essential to contextualize the findings. On the other hand, the presence of gray matter hyperintensities in a younger individual may raise suspicion of a different underlying pathology, such as inflammatory conditions or neurodegenerative diseases. The age of the patient and the clinical history play a crucial role in interpreting hyperintensities [1].

Additional imaging sequences: To enhance the differentiation of white matter and gray matter hyperintensities, advanced imaging sequences, such as susceptibility-weighted imaging (SWI) and diffusion-weighted imaging (DWI), can be employed. SWI is particularly useful in visualizing blood products, microbleeds, and calcifications, aiding in identifying potential causes of hyperintensities. DWI, on the other hand, measures the diffusion of water molecules in tissues, providing insights into tissue microstructure. These additional sequences can provide valuable complementary information that assists in characterizing hyperintensities and determining their underlying etiology. Integrating such advanced sequences into the imaging protocol enhances diagnostic precision and the ability to distinguish between different types of hyperintensities [23].

Neuroimaging techniques for assessing T2-weighted hyperintensities

MRI Sequences and Parameters

T2-weighted imaging: T2-weighted MRI sequences are the primary and foundational imaging modality for visualizing hyperintensities in neuroimaging. These sequences emphasize differences in tissue water content, making them well-suited for detecting regions with increased water content, such as those containing hyperintensities. The elevated signal intensity of hyperintensities in T2-weighted images contrasts with the surrounding brain tissue, allowing for their identification and assessment. T2-weighted sequences are particularly effective in highlighting the presence of hyperintensities in the brain and serve as a starting point for their characterization [24].

Fluid-attenuated inversion recovery (FLAIR): FLAIR sequences are instrumental in improving the visualization of WMH. FLAIR sequences are designed to suppress the cerebrospinal fluid (CSF) signal, enhancing the contrast between hyperintensities and the surrounding brain tissue. By reducing the influence of the CSF signal, FLAIR images provide a more precise depiction of WMH, which can be challenging to differentiate from normal brain tissue in conventional T2-weighted images [25].

T1-weighted imaging: T1-weighted images are valuable for providing anatomical reference and spatial localization of hyperintensities. While T1-weighted sequences may not directly highlight the hyperintensities themselves, they are essential for co-registration with T2-weighted or FLAIR images, aiding in precise localization and ensuring that the identified hyperintensities are accurately mapped onto the brain’s anatomical structures. T1-weighted imaging contributes to the overall spatial context of hyperintensity assessment [26].

High-resolution imaging: High-resolution MRI sequences are employed when fine details of hyperintensities are required, such as when assessing smaller lesions or conducting research studies. These sequences offer improved image clarity and the ability to capture subtle variations in hyperintensity characteristics. High-resolution imaging is particularly valuable in research applications, where precise measurements and detailed assessment of hyperintensities are critical for the study’s objectives [27].

Multi-contrast imaging: Employing multiple MRI sequences, including T1-weighted, T2-weighted, and FLAIR, can provide a more comprehensive assessment of hyperintensities. Each sequence contributes unique information about the location, morphology, and properties of the hyperintensities. Integrating these multiple imaging modalities allows healthcare professionals to understand the hyperintensities holistically, aiding in their characterization and precise diagnosis. The combination of multi-contrast imaging enhances the sensitivity and specificity of hyperintensity assessment, mainly when dealing with complex cases or when differentiation between different types of lesions is challenging [27].

Image Acquisition and Preprocessing

Image resolution: High spatial resolution is critical for accurately visualizing hyperintensities on MRI scans. It enables the differentiation and characterization of smaller lesions and fine structural details. Achieving high spatial resolution involves acquiring MRI images with thin slices and small voxel sizes. Thin slices provide detailed cross-sectional images of the brain, while small voxel sizes allow for precise localization and differentiation of hyperintensities within the brain tissue. High spatial resolution is precious when assessing small or subtle hyperintensities that may be overlooked at lower resolutions [28].

Motion correction: Minimizing motion artifacts during image acquisition is essential for obtaining clear and diagnostically accurate MRI images. Motion artifacts can distort the appearance of hyperintensities and reduce image quality. Various motion correction techniques are employed, including prospective and retrospective image registration. Prospective motion correction involves adjusting the imaging parameters in real time to compensate for patient motion during the scan. Retrospective image registration aligns images post-acquisition to correct for any motion that occurred during the scan. These techniques help ensure that the images accurately represent hyperintensities, improving diagnostic reliability [29].

Signal intensity normalization: Signal intensity normalization is a critical step in MRI analysis, particularly when comparing images from different scans or patients. It involves standardizing the signal intensity values to account for variations in acquisition parameters, scanner hardware, and patient characteristics. Signal intensity normalization ensures that hyperintensities are quantitatively and qualitatively comparable between different scans, enabling meaningful comparative analysis. Normalization procedures maintain consistency in image interpretation and research studies [30].

Noise reduction: MRI images can be affected by noise, which can obscure hyperintensities and reduce image clarity. Noise reduction techniques, such as image denoising, enhance the quality of MRI images. These techniques filter out unwanted noise while preserving important image features, resulting in cleaner and more visually interpretable images. Reducing noise is particularly important when assessing hyperintensities, as it helps to distinguish these regions from noise artifacts and improves the accuracy of their visualization and characterization [31].

Co-registration: Co-registration involves aligning MRI images with other imaging modalities, such as anatomical or functional images, to facilitate precise localization and correlation of hyperintensities with specific brain regions or functional areas. This process enables the integration of structural and functional information, enhancing the understanding of the relationship between hyperintensities and brain function. Co-registration is particularly valuable in research and clinical applications where a comprehensive view of hyperintensities’ impact on brain function or anatomy is desired. It enables healthcare professionals and researchers to explore the spatial relationships between hyperintensities and other brain structures, leading to a more holistic assessment [32].

Quantitative and Semi-Quantitative Analysis Methods

Volumetric analysis: Volumetric analysis involves calculating the volume or size of hyperintensities within the brain. This analysis is precious for assessing the extent and progression of lesions over time. In diseases like MS, quantifying hyperintensity volumes in serial MRI scans can provide critical information about disease activity and the response to treatment. Changes in lesion volume are often used as outcome measures in clinical trials and longitudinal studies, enabling healthcare professionals to monitor disease evolution and therapeutic efficacy [33].

Intensity histogram analysis: Intensity histogram analysis involves constructing histograms of signal intensity values within hyperintensities. These histograms provide insights into the heterogeneity of hyperintensities and help characterize their distribution. Analyzing signal intensity distributions within hyperintensities can reveal patterns and variations, which may indicate different underlying pathologies. This approach is beneficial for investigating the heterogeneity of hyperintensities and can aid in differentiating between various lesion types or stages [34].

Region of interest (ROI) analysis: ROI analysis involves drawing regions of interest around hyperintensities to perform specific measurements. These measurements include calculating the mean signal intensity within the ROI or extracting texture features that describe the spatial arrangement and patterns of intensity values. ROI analysis allows for a more detailed and focused characterization of hyperintensities, enabling the assessment of their specific properties and features. It is often used in research and clinical applications to better understand hyperintensity characteristics [35].

DWI: Combining DWI with T2-weighted images is a valuable approach for assessing the diffusion characteristics of hyperintensities. DWI provides information about the movement of water molecules within tissues, allowing for the differentiation of tissue types based on their diffusion properties. This can aid in distinguishing between hyperintensities with different underlying pathologies, such as ischemia, inflammation, or necrosis. The integration of DWI enhances diagnostic precision and the ability to differentiate between hyperintensities with distinct etiologies [36].

Machine learning and artificial intelligence (AI): Machine learning and AI algorithms have revolutionized the field of hyperintensity analysis by automating lesion detection, segmentation, and classification. These algorithms use trained models to identify and characterize hyperintensities, improving the efficiency and accuracy of assessments. AI-based approaches can analyze large datasets rapidly and consistently, reducing inter-rater variability and increasing the objectivity of hyperintensity interpretation. They have become indispensable clinical practice and research tools, enabling more efficient and precise hyperintensity analysis [37]. The workflow visualization in machine learning applications for automating lesion detection, segmentation, and classification of hyperintensities in MRI data is presented in Table 1.

**Table 1:** Workflow visualization in machine learning applications for automating lesion detection, segmentation, and classification of hyperintensities in MRI data MRI, magnetic resonance imaging

Workflow step	Description
Data preprocessing	Preprocess MRI data by performing image registration, normalization, and noise reduction.
Training data collection	Collect annotated MRI scans with hyperintensities labeled by experts for model training.
Feature extraction	Extract features from MRI data to represent the characteristics of hyperintensities.
Model training	Train machine learning models using collected data and extracted features.
Lesion detection	Utilize trained models to detect hyperintensities in MRI scans.
Segmentation	Segment hyperintensities from surrounding brain tissue using machine learning algorithms.
Classification	Classify hyperintensities into different categories or disease states based on extracted features.
Post-processing	Filter false positives, refine lesion boundaries, and validate results using validation metrics.
Output for clinical interpretation	Present the final results, including detected, segmented, and classified hyperintensities, for clinical interpretation.
Integration into the clinical workflow	Integrate automated tools into the clinical workflow for use by radiologists and clinicians.

Clinical applications

Diagnostic Value in Neurological Diseases

MS: T2-weighted hyperintensities are integral to diagnosing and managing MS. These hyperintensities, often seen in the white matter, are a hallmark feature of the disease. These lesions’ distribution, size, and enhancement patterns are considered vital diagnostic criteria. They play a critical role in assessing disease activity, monitoring lesion evolution, and evaluating the effectiveness of disease-modifying therapies. Serial imaging and quantitative analysis of hyperintensity burden aid in clinical decision-making and treatment planning for patients with MS [38].

Vascular disorders: Hyperintensities on T2-weighted MRI scans are instrumental in diagnosing and evaluating vascular disorders, including stroke and small vessel disease. They can highlight areas of ischemia and infarction, providing insights into the extent and severity of vascular pathology. These hyperintensities aid in the differentiation of acute from chronic infarcts and guide treatment decisions. In conditions like small vessel disease, hyperintensities in the deep white matter indicate microvascular changes and contribute to assessing cerebrovascular risk factors [39].

Dementia: WMH, frequently observed in patients with Alzheimer’s disease and vascular dementia, are valuable markers for differentiating between various forms of dementia and assessing their underlying causes. The presence and extent of these hyperintensities are associated with cognitive decline and contribute to the characterization of dementia subtypes. Their location and burden aid in understanding the impact of white matter changes on cognitive function and guiding therapeutic interventions in dementia management [18].

Brain tumors: Hyperintensities in T2-weighted images indicate brain tumors and the associated peritumoral edema. These hyperintensities are instrumental in the localization and assessment of the extent of the tumor. The characteristics of hyperintensities, such as their enhancement patterns on contrast-enhanced scans, contribute to differentiated tumor types and assist in surgical planning. Monitoring changes in hyperintensity size and distribution over time is crucial for assessing treatment response and tumor progression [16].

Inflammatory and infectious conditions: T2-weighted hyperintensities are observed in various brain inflammatory and infectious conditions, including encephalitis. Their presence on MRI scans aids in early diagnosis and guides appropriate treatment strategies. The distribution and characteristics of hyperintensities provide insights into the extent of inflammation and the affected brain regions. Identifying hyperintensities in these conditions is critical for initiating timely interventions to manage the underlying pathology and improve patient outcomes [40].

Prognostic Implications

Vascular events: Hyperintensities on MRI scans in the context of vascular events, such as stroke and small vessel disease, can serve as important prognostic indicators. The extent and severity of hyperintensities in these conditions can correlate with long-term functional outcomes. For instance, the volume and distribution of hyperintensities in a stroke patient’s brain may provide insights into the extent of tissue damage and the potential for recovery. Moreover, hyperintensities can indicate ongoing vascular pathology, which may increase the risk of recurrent vascular events. Identifying and quantifying hyperintensities in these cases is essential for assessing the prognosis and tailoring rehabilitation and secondary prevention strategies [1].

MS: Monitoring the evolution of hyperintensities in patients with MS is crucial for assessing disease activity and guiding treatment decisions. Hyperintensities in MS represent demyelination and inflammation, and their presence and changes over time are critical markers of disease progression. An increase in lesion burden, as evidenced by the development of new hyperintensities or the enlargement of existing ones, may signal disease activity and a poorer prognosis. Serial MRI scans are routinely used to track the evolution of hyperintensities and guide the selection of disease-modifying therapies. Accurately assessing hyperintensity characteristics and changes over time is essential for optimizing MS management [38].

Dementia: In individuals with dementia, the presence and progression of hyperintensities, mainly WMH, can predict cognitive decline and the severity of the condition. These hyperintensities are often associated with vascular contributions to cognitive impairment and dementia. Monitoring the size and distribution of hyperintensities over time can provide valuable insights into the rate of cognitive deterioration and help differentiate between various forms of dementia. Understanding the relationship between hyperintensities and cognitive function is critical for caregivers and healthcare providers to plan appropriate interventions and support for individuals with dementia [41].

Brain tumors: Hyperintensities in the context of brain tumors are instrumental in informing the prognosis and guiding treatment strategies for patients. The characteristics of these hyperintensities, such as their size, location, and enhancement patterns on contrast-enhanced scans, provide valuable information about the tumor’s nature and aggressiveness. For example, more extensive hyperintensities may indicate a higher tumor burden or peritumoral edema, potentially influencing the patient’s prognosis. Monitoring changes in hyperintensity size and distribution over time is essential for assessing the response to treatment and the risk of tumor progression. Accurate characterization of hyperintensities in brain tumor patients is fundamental for making informed clinical decisions and optimizing patient care [42].

Monitoring Disease Progression and Treatment Response

MS: Regular MRI scans are a cornerstone of MS management, as they monitor the evolution of hyperintensities in MS patients. These hyperintensities represent areas of demyelination and inflammation in the central nervous system. Serial MRI scans track lesion size, count, and distribution changes over time. These changes provide critical information about disease activity and response to treatment. An increase in lesion burden, demonstrated by the development of new hyperintensities or the enlargement of existing ones, may signal disease progression and suggest a need for adjustments to disease-modifying therapies. Conversely, a reduction in hyperintensity burden may indicate treatment efficacy. Regular MRI monitoring in MS is essential for assessing the effectiveness of therapeutic interventions and optimizing patient care [22].

Stroke and vascular diseases: Serial MRI imaging is invaluable in assessing the impact of interventions in stroke and vascular diseases. For instance, in the context of an ischemic stroke, MRI scans performed at different time points can reveal the evolution of hyperintensities and tissue damage. This information helps healthcare providers evaluate the effects of treatments like thrombolytic therapy and make informed decisions about patient management. Serial imaging can show the extent of lesion recovery or, conversely, the progression of infarcted tissue. It plays a crucial role in assessing the efficacy of interventions and guiding the rehabilitation process, ultimately improving patient outcomes [43].

Brain tumors: Imaging is fundamental for assessing the response of brain tumors to various treatment modalities, including radiation therapy, chemotherapy, and surgical interventions. Hyperintensity patterns on MRI scans indicate tumor presence, extent, and peritumoral edema. Serial MRI scans conducted before, during, and after treatment can demonstrate changes in hyperintensity characteristics. For example, a decrease in hyperintensity size or enhancement following treatment suggests a positive response, while an increase may indicate disease progression or treatment resistance. These imaging findings inform clinical decisions and help guide therapeutic strategies, such as modifying treatment regimens or planning surgical interventions, to optimize patient care and improve tumor management [44].

Infectious diseases: Imaging is vital in assessing infectious diseases affecting the central nervous system, such as encephalitis. MRI scans enable healthcare providers to determine the response to antiviral or antibiotic treatments. Changes in hyperintensity characteristics, including size, distribution, and enhancement patterns, are used to gauge treatment efficacy. A reduction in hyperintensity size and the resolution of inflammation on MRI may indicate a positive response to treatment and guide decisions regarding the duration and modification of therapy. Serial imaging in infectious diseases ensures that patients receive appropriate and timely interventions, ultimately improving the chances of recovery and reducing the risk of complications [45].

Challenges and pitfalls in T2-weighted hyperintensities assessment

Image Artifacts

Motion artifacts: Motion artifacts are a common challenge in MRI imaging, as patient motion during the scan can introduce blurring or ghosting. These artifacts can obscure hyperintensities, making identifying and characterizing them difficult. To mitigate motion artifacts, various techniques are employed. These include motion correction methods that adjust imaging parameters in real time to compensate for patient movement during the scan. Additionally, restraint devices or strategies to minimize patient motion, such as providing clear instructions and support, are implemented to improve image quality and reduce motion-related distortions [46].

Susceptibility artifacts: Susceptibility artifacts arise due to differences in magnetic susceptibility between tissues and can cause distortions in MRI images, potentially leading to false hyperintensity appearances. These artifacts are particularly relevant in regions near air-tissue interfaces, such as the sinuses or the base of the skull. In these areas, susceptibility artifacts can affect the interpretation of hyperintensities, as they may produce exaggerated signal changes. Techniques like SWI address susceptibility artifacts and provide a more accurate representation of hyperintensities in regions prone to susceptibility-related distortions [47].

Scanner and hardware artifacts: Variations in MRI scanner hardware, including gradient nonlinearities and radiofrequency coil characteristics, can introduce geometric distortions and intensity variations in images. These artifacts can impact the assessment of hyperintensities by altering their appearance and location. Calibrating and standardizing scanner hardware and conducting regular quality control procedures are essential for minimizing these artifacts. Additionally, post-processing techniques can be employed to correct geometric distortions caused by hardware-related artifacts [48].

Chemical shift artifacts: Chemical shift artifacts can lead to misinterpretations of MRI images, particularly in regions with fat-water interfaces. These artifacts occur due to the differing resonant frequencies of hydrogen atoms in fat and water molecules. In areas with fat-water interfaces, chemical shift artifacts may create the appearance of hyperintensities if not adequately accounted for during image analysis. Advanced MRI sequences, such as Dixon imaging, are designed to address chemical shift artifacts and improve the accuracy of hyperintensity assessment by enabling the separation of fat and water signals, thus reducing potential misinterpretations in these regions [49].

Inter-Rater Variability

Subjectivity: The interpretation of T2-weighted hyperintensities in MRI images can be subject to inter-rater variability, where different radiologists or researchers may provide varying assessments of the same images. This variability arises from differences in experience, training, and personal judgment. For example, less-experienced individuals may need help with the nuanced differentiation of hyperintensities, leading to discrepancies in their identification and characterization. To address this challenge, efforts should be made to establish consensus and promote consistent interpretation among raters. Peer review and consensus meetings involving multiple experts can help reduce subjectivity and improve the reliability of hyperintensity assessment [50].

Lack of standardization: The need for standardized criteria for assessing T2-weighted hyperintensities can exacerbate variability in their interpretation. With clear guidelines and criteria for hyperintensity assessment, each rater may develop their own approach, leading to consistency in the characterization of lesions. Establishing standardized protocols and criteria for hyperintensity assessment is crucial to reduce subjectivity and ensure that assessments are conducted consistently across clinical and research settings. Standardization efforts may involve the development of clinical practice guidelines or research protocols that define criteria for lesion identification, classification, and reporting [51].

Intra-rater variability: In addition to inter-rater variability, even experienced raters may exhibit intra-rater variability when assessing the same images at different times. This variability may result from differences in the rater’s mental state, fatigue, or evolving interpretation patterns. Standardized protocols and repeated training are essential to mitigate intra-rater variability. Regular calibration and quality control measures can help maintain consistency in hyperintensity assessment. Ongoing education and refresher training for raters, especially in research or clinical trial settings, can also contribute to reducing intra-rater variability and enhancing the reproducibility of assessments [52].

Limitations of Current Techniques

Limited specificity: T2-weighted images, while valuable for identifying hyperintensities, may lack specificity in determining the precise underlying cause of these lesions. Additional imaging sequences, such as contrast-enhanced images, DWI, or spectroscopy, along with clinical information and, in some cases, biopsy or pathology reports, are often needed to establish a definitive diagnosis. These complementary data sources provide a more comprehensive understanding of hyperintensity and its etiology, allowing healthcare providers to make accurate diagnostic decisions [53].

Incomplete characterization: T2-weighted images primarily offer information about hyperintensity location and signal intensity. However, they may need to completely characterize the lesion’s nature or stage. Advanced imaging techniques, such as contrast-enhanced MRI, may be required to assess factors like lesion enhancement, which can indicate active inflammation or neovascularization. Other imaging modalities or ancillary tests, including positron emission tomography (PET) or CSF analysis, may be necessary to obtain a more detailed characterization of the lesion, its composition, and its functional properties [4].

Mixed pathology: Hyperintensities often result from a combination of underlying pathological processes. This complexity can make attributing the lesions to a single cause challenging. For example, lesions may exhibit both inflammatory and ischemic components or represent a combination of vascular and neurodegenerative changes. The coexistence of multiple pathologies within the same lesion can complicate the interpretation and diagnosis, necessitating a comprehensive approach considering all potential contributing factors [54].

Limited temporal information: T2-weighted imaging provides a static snapshot of the brain at a specific moment. Dynamic changes over time, such as lesion progression or regression, may not be fully assessed without longitudinal imaging. Monitoring changes in hyperintensity characteristics across multiple time points using serial MRI scans is crucial for evaluating disease evolution, treatment response, and the assessment of lesion dynamics. Combining T2-weighted images with serial imaging over different time intervals provides a more comprehensive view of hyperintensity behavior [4].

Lesion heterogeneity: Hyperintensities within the same patient can exhibit significant size, intensity, and distribution heterogeneity. This heterogeneity further complicates their assessment and interpretation. Lesions with varying characteristics may represent different underlying pathologies or stages of disease. Identifying and characterizing these diverse lesions within a single patient requires a nuanced approach that considers the potential contributing factors to the observed heterogeneity, emphasizing the importance of multimodal imaging and clinical correlation [55].

Emerging technologies and advancements

Novel MRI Techniques

SWI: SWI is an advanced MRI technique that significantly enhances the visibility of structures with differing magnetic susceptibilities, such as blood and iron deposits. SWI provides superior contrast and sensitivity for detecting small lesions, including microbleeds and calcifications. These subtle abnormalities may not be as readily apparent on conventional T2-weighted images, making SWI an invaluable tool for assessing T2-weighted hyperintensities, especially in cases of cerebrovascular diseases and traumatic brain injury. The enhanced visualization of microbleeds and iron-related changes can contribute to a more accurate diagnosis and a better understanding of the underlying pathology [56].

Diffusion tensor imaging (DTI): DTI is a specialized MRI technique that measures the diffusion of water molecules in tissues, providing valuable insights into the microstructural integrity of white matter fiber tracts. DTI is particularly valuable for assessing WMH and their impact on the brain’s structural connectivity. DTI can identify abnormalities in white matter tracts associated with hyperintensities by characterizing the directional movement of water molecules. It helps quantify changes in the integrity of fiber bundles, making it a powerful tool for understanding the functional consequences of hyperintensities, especially in conditions like MS, where white matter damage is a key feature [57].

High-field MRI: Using higher magnetic field strengths, such as 3 Tesla (3T) or 7 Tesla (7T) MRI, offers several advantages for assessing T2-weighted hyperintensities. High-field MRI systems provide improved image resolution and a higher signal-to-noise ratio, which allows for a more detailed and precise assessment of hyperintensities. This enhanced imaging quality is particularly beneficial when investigating small lesions, subtle changes, or lesions in anatomically challenging regions. The increased signal-to-noise ratio in high-field MRI not only improves lesion visibility but also enhances the ability to differentiate between various tissue types and characteristics, ultimately contributing to a more accurate diagnosis and characterization of hyperintensities. High-field MRI is especially valuable for research applications and investigations requiring the highest image quality [48].

AI and Machine Learning in T2-Weighted Hyperintensities Analysis

Automated detection and segmentation: Machine learning algorithms, particularly convolutional neural networks, have made significant advancements in automating the detection and segmentation of T2-weighted hyperintensities in MRI images. These algorithms can analyze large datasets of medical images and accurately identify the location and extent of hyperintensities, reducing the need for time-consuming manual assessment. Automated detection and segmentation not only save time but also improve the reproducibility of hyperintensity assessment, minimizing inter-rater variability. This technology is precious in clinical practice, research, and large-scale studies where efficient and consistent lesion quantification is essential [58].

Quantitative analysis: Machine learning models can extract a wide range of quantitative features from T2-weighted images, allowing for a more in-depth assessment of hyperintensities. These features may include texture analysis, fractal analysis, and intensity-based metrics, among others. Quantitative analysis goes beyond visual inspection and objectively measures hyperintensity characteristics, such as heterogeneity, shape, and spatial distribution. These metrics can help differentiate between lesion types, monitor changes over time, and contribute to a better understanding of the underlying pathology. Additionally, quantitative analysis is precious for research applications and may uncover subtle patterns that are not readily apparent through visual assessment alone [59].

Predictive modeling: Machine learning models can be trained to predict various clinical outcomes based on T2-weighted hyperintensities and other clinical data. For example, AI models can predict disease progression, treatment response, and patient outcomes. By incorporating hyperintensity characteristics into predictive models, healthcare providers can personalize treatment strategies and make more informed clinical decisions. This approach can improve patient care by tailoring interventions to individual needs, optimizing treatment efficacy, and enhancing long-term outcomes. Predictive modeling also offers opportunities for early intervention and proactive patient management in conditions like MS and stroke [60].

Future Prospects and Research Directions

Multimodal integration: The future of neuroimaging lies in the integration of multiple imaging modalities, such as functional MRI, PET, and DWI, in combination with T2-weighted images. This multimodal approach offers a more comprehensive understanding of brain pathology, combining information about anatomical structures, functional connectivity, and metabolic activity. Integrating T2-weighted imaging with other modalities allows researchers and clinicians to assess the relationship between hyperintensities and brain function, enhancing diagnostic accuracy and the ability to identify the underlying mechanisms of neurological disorders [61].

Longitudinal studies: Longitudinal studies that track changes in T2-weighted hyperintensities over time are critical for gaining insights into disease progression and the impact of interventions. These studies provide valuable data for understanding the natural history of neurological conditions and evaluating the effectiveness of therapeutic approaches. By monitoring the evolution of hyperintensities, researchers can identify predictive biomarkers, refine treatment strategies, and assess the long-term effects of interventions. Longitudinal research is crucial for improving patient care and advancing our knowledge of disease dynamics [62].

Biomarker development: T2-weighted hyperintensities are being actively explored as potential biomarkers for various neurological diseases. Researchers are working to identify specific patterns and features within these hyperintensities that correlate with disease subtypes, stages, and clinical outcomes. Developing robust biomarkers based on T2-weighted imaging can facilitate earlier diagnosis, provide valuable prognostic information, and guide treatment decisions. These biomarkers may also serve as endpoints in clinical trials, enabling a more precise evaluation of the efficacy of therapeutic interventions [63].

Therapeutic targets: Investigating the underlying pathophysiology of T2-weighted hyperintensities and their relationship with neurological diseases can lead to the discovery of novel therapeutic targets. Understanding the mechanisms driving the formation and progression of hyperintensities can open avenues for targeted interventions. These therapies may aim to reduce lesion burden, prevent their formation, or promote lesion resolution, ultimately revolutionizing treatment approaches for conditions like MS, vascular dementia, and stroke [64].

Enhanced visualization: Ongoing developments in image post-processing, visualization techniques, and three-dimensional reconstructions aim to improve the visualization of T2-weighted hyperintensities. Enhanced visualization tools can aid in the precise localization and characterization of hyperintensities, making subtle changes more readily apparent. These innovations contribute to the accuracy of lesion identification and support more detailed analyses, benefiting clinical practice and research endeavors. Improved visualization methods are particularly relevant in cases where hyperintensities are small or located in complex anatomical regions [65].

## Conclusions

Our comprehensive review has unveiled the pivotal role of T2-weighted hyperintensities in neuroimaging. These areas of heightened signal intensity on MRI images are essential for the diagnosis, prognosis, and monitoring of a wide range of neurological diseases. They are particularly significant in conditions such as MS, vascular disorders, dementia, and brain tumors, where their characteristics and location can hold crucial diagnostic and therapeutic implications. Our exploration also highlighted the evolution of neuroimaging techniques, including advanced MRI modalities and the integration of AI, which are poised to revolutionize the assessment of hyperintensities. As we look to the future, the development of biomarkers, personalized medicine, and enhanced imaging technology promises to further enhance the accuracy and efficacy of patient care, offering the potential to target interventions with precision. The overall significance of T2-weighted hyperintensities in neuroimaging cannot be overstated, as they continue to shape the landscape of neurological diagnosis and research, facilitating a deeper understanding of complex neurological conditions and paving the way for more informed and effective clinical practice.
